# Survival and Reproduction of *Myxobolus cerebralis*-Resistant Rainbow Trout Introduced to the Colorado River and Increased Resistance of Age-0 Progeny

**DOI:** 10.1371/journal.pone.0096954

**Published:** 2014-05-08

**Authors:** Eric R. Fetherman, Dana L. Winkelman, Melinda R. Baerwald, George J. Schisler

**Affiliations:** 1 Colorado Parks and Wildlife, Fort Collins, Colorado, United States of America; 2 US Geological Survey, Colorado Cooperative Fish and Wildlife Research Unit, Department of Fish, Wildlife and Conservation Biology, Colorado State University, Fort Collins, Colorado, United States of America; 3 Genomic Variation Laboratory, Department of Animal Science, University of California Davis, Davis, California, United States of America; Auburn University, United States of America

## Abstract

*Myxobolus cerebralis* caused severe declines in rainbow trout populations across Colorado following its introduction in the 1980s. One promising approach for the recovery of Colorado’s rainbow trout populations has been the production of rainbow trout that are genetically resistant to the parasite. We introduced one of these resistant crosses, known as the GR×CRR (cross between the German Rainbow [GR] and Colorado River Rainbow [CRR] trout strains), to the upper Colorado River. The abundance, survival, and growth of the stocked GR×CRR population was examined to determine if GR×CRRs had contributed offspring to the age-0 population, and determine whether these offspring displayed increased resistance and survival characteristics compared to their wild CRR counterparts. Apparent survival of the introduced GR×CRR over the entire study period was estimated to be 0.007 (±0.001). Despite low survival of the GR×CRRs, age-0 progeny of the GR×CRR were encountered in years 2008 through 2011. Genetic assignments revealed a shift in the genetic composition of the rainbow trout fry population over time, with CRR fish comprising the entirety of the fry population in 2007, and GR-cross fish comprising nearly 80% of the fry population in 2011. A decrease in average infection severity (myxospores fish^−1^) was observed concurrent with the shift in the genetic composition of the rainbow trout fry population, decreasing from an average of 47,708 (±8,950) myxospores fish^−1^ in 2009 to 2,672 (±4,379) myxospores fish^−1^ in 2011. Results from this experiment suggest that the GR×CRR can survive and reproduce in rivers with a high prevalence of *M. cerebralis*. In addition, reduced myxospore burdens in age-0 fish indicated that stocking this cross may ultimately lead to an overall reduction in infection prevalence and severity in the salmonid populations of the upper Colorado River.

## Introduction

Extirpations of wild salmonid populations have been caused by a variety of factors and have led to a focus on captive breeding (i.e., hatcheries) to sustain or reintroduce populations [1]–[4]. However, successful reintroduction attempts using captive-reared salmonids usually involve mitigating or removing the factors responsible for the original extirpation [5]. For instance, artificial liming has been used to reduce river acidification in Norway and has aided in successful reintroduction of Atlantic salmon (*Salmo salar*) [1]. Greenback cutthroat trout (*Oncorhynchus clarki stomias*) have also been successfully reintroduced in streams with suitable habitat that are protected from reinvasion by other invasive trout species [6]. However, when factors causing extirpations have not been fully mitigated prior to reintroduction, stocking has generally been unsuccessful [5].

In Colorado, introduction of *Myxobolus cerebralis*, the parasite responsible for salmonid whirling disease, caused the extirpation of wild rainbow trout (*Oncorhynchus mykiss*) populations from many of the state’s rivers. Natural recruitment of wild rainbow trout has been almost nonexistent in these affected rivers since the establishment of *M. cerebralis* in the late 1980s [7]. Unlike extirpations caused by factors that could potentially be mitigated or reversed, pathogens such as *M. cerebralis* cannot presently be removed once introduced into an ecosystem. However, disruption of the parasite’s life cycle has been attempted both through habitat manipulation to reduce populations of the intermediate oligochaete host (*Tubifex tubifex*) and through introduction of resistant lineages of *T. tubifex*. Neither approach has been completely successful [8]. One promising approach for the recovery of Colorado’s rainbow trout populations has been the production of rainbow trout that are genetically resistant to the parasite. To produce a suitable rainbow trout for reintroduction, management and research in Colorado have focused on using crosses between resistant, hatchery-derived rainbow trout and wild rainbow trout strains [9].

Rainbow trout are native to western North America, but have been transported around the world for use in aquaculture and to establish wild trout fisheries [10]. The German Rainbow (GR) is a hatchery-derived rainbow trout strain that was exposed to *M. cerebralis* for decades in a hatchery in Germany [11]. Although the GR strain can be infected with *M. cerebralis*, parasite burdens are usually low [9], [11], [12] and the GR strain is known to survive and reproduce in the presence of, and when infected with, *M. cerebralis*. Low parasite burdens and the strain’s ability to persist when exposed to *M. cerebralis* have been termed “resistance,” and this resistance is presumed to be a result of long-term exposure to the parasite over multiple generations [11]. Despite the resistance seen in the GR strain, its survival and viability in the wild was uncertain due to the strain’s history of domestication [9]. Therefore, the GR strain was experimentally crossed with the Colorado River Rainbow (CRR) [9], [12], [13], a wild rainbow trout strain that had been widely stocked in Colorado and was used to establish many naturally reproducing wild rainbow trout fisheries prior to the introduction of *M. cerebralis* [14].

Intermediate crosses of the two strains have been rigorously evaluated. Laboratory experiments showed that the first filial (F_1_) generational cross between the two strains (termed the GR×CRR) exhibited resistance characteristics similar to those of the GR strain [9], [12], and was capable of attaining critical swimming velocities similar to those of the CRR strain [13]. It was suggested that the GR×CRR cross may be the best candidate for reintroducing rainbow trout populations; however, its utility needed to be evaluated in a natural setting [12]. Overall, we wanted to evaluate the performance of GR×CRR that were stocked into the upper Colorado River in an attempt to reintroduce a self-sustaining population in the presence of *M. cerebralis*. The objectives of our study were to examine the abundance, survival, growth, and reproduction of the stocked GR×CRR population in the upper Colorado River and determine if their offspring displayed increased resistance characteristics compared to their wild CRR counterparts.

## Methods

### Ethics Statement

The field sampling protocol for this study was approved by the Institutional Animal Care and Use Committee at Colorado State University (Protocol Number: 10-1957A). Proper settings for the electrofishing units, determined by conductivity of the river and size of fish being sampled, were used to minimize fish injury. Fish were held for the shortest amount of time possible to examine fish for individual marks, collect length and weight data, and collect genetic samples, to minimize suffering.

### Site Description

**Figure 1:**
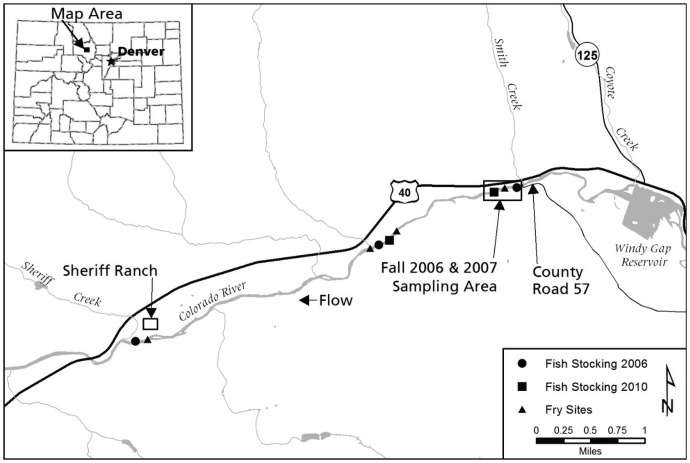
The upper Colorado River study site. The 4.2-57 at the upstream end and Sheriff Creek at the downstream end. Locations in which fish were sampled in 2006 and 2007 (box), fry were sampled in all years of the study (triangles), and fish were stocked in 2006 (circles) and 2010 (squares) are shown.

The 4.2 km upper Colorado River study site is situated approximately 1.6 km downstream of Windy Gap Reservoir and 3.2 km upstream of the town of Hot Sulphur Springs in Grand County, Colorado (Figure 1). Flows in this section are partially regulated by Windy Gap dam, with a mean annual discharge of 7.2 cubic meters per second (cms), ranging from a mean of 2.2 cms in the winter to 22.5 cms during peak flows [15]. Minimum and maximum discharge values, which were used as predictor variables affecting adult GR×CRR survival, were obtained from a USGS stream gauge located at the upstream end of the study section near the CR-57 bridge (Figure 1). Temperatures in the upper Colorado River range from 3.4°C in the winter to 16.2°C in the summer, with a mean annual temperature of 10.7°C [15]. The study section is on private land, primarily managed for cattle grazing; however, land owners allow private fishing access.

Prior to the introduction of *M. cerebralis* in the upper Colorado River, adult CRR had an average abundance of 428 fish km^−1^ and adult brown trout averaged 239 fish km^−1^ [7], resulting in a ratio of rainbow trout to brown trout of 2∶1. Rainbow trout fry abundance ranged from 5,600 to 8,400 fry km^−1^of stream bank and brown trout fry ranged from 2,600 to 5,700 fry km^−1^ [14]. Traditionally, eggs were harvested from this wild CRR brood stock, reared in state hatcheries, and used to stock many rivers across the state.

*Myxobolus cerebralis* was unintentionally introduced to the upper Colorado River in the 1980s when privately-reared rainbow trout previously exposed to *M. cerebralis* were stocked into three private water bodies located upstream of Windy Gap Reservoir. Fish below Windy Gap Reservoir tested positive for *M. cerebralis* in 1988, and a subsequent decline in the younger age classes of rainbow trout was observed in the early 1990s [16]. While several reasons for the declines were investigated [17]–[19], exposure to *M. cerebralis* was determined to be the primary cause for the disappearance of the younger age classes [7]. In an effort to restore the rainbow trout fishery, tens of thousands of CRR were stocked annually between 1994 and 2008. Despite these repeated stocking efforts, the CRR exhibited low survival and little recruitment success, resulting in rainbow trout abundances that were approximately 90% lower than those observed prior to the establishment of *M. cerebralis* [16]. The upper Colorado River below Windy Gap Reservoir continues to be one of the rivers with the highest prevalence of *M. cerebralis* infection in the state.

### Rainbow Trout Stocking

The first introduction of *M. cerebralis*-resistant rainbow trout to the upper Colorado River occurred on June 2, 2006, with an introduction of 3,000 GR×CRRs. Prior to being stocked, each fish was tagged with an individually numbered fine-filament Floy tag, secondarily adipose clipped for identification in the event of tag loss, and measured to the nearest mm; fish averaged 238 (±23) mm in total length (TL). Larger rainbow trout were used in the introduction because they were 1) less susceptible to *M. cerebralis* infection [20], and 2) less susceptible to brown trout predation. Fish were distributed throughout the study section, with approximately 1,250 fish stocked at the upstream end of the section, 1,100 stocked in the middle of the section, and 650 stocked at the downstream end of the section (Figure 1).

An additional introduction occurred in June 2010, with 2,000 GR×CRRs averaging 172 (±18) mm TL stocked at the upstream end and middle of the section (1,000 fish in each location; Figure 1). These fish were similarly tagged with individually numbered Floy tags and measured to the nearest mm prior to stocking. Only one sampling occasion occurred following this introduction of GR×CRRs in 2010, and as a result, survival was not estimable for these fish. However, these fish contributed to adult fish population abundance estimates in 2011 and potentially contributed offspring produced during the study. Therefore, survival and growth analyses regarding the adult rainbow trout population are performed using only data collected from the group of GR×CRRs introduced to the upper Colorado River in 2006, but abundance estimates include fish introduced in 2006 and 2010.

### Adult Rainbow Trout Population

#### Abundance estimation

A two-pass, mark-recapture electrofishing effort, with a minimum of one day between passes to allow for the redistribution of marked fish, was used to estimate abundance of the adult rainbow trout population in the upper Colorado River in the spring of 2008, 2009, 2010, and 2011. Two raft-mounted electrofishing units were used to complete the estimates, with one raft covering each half of the river. Fish encountered on both the mark and recapture passes were processed approximately every 0.8 km and returned to the river following processing. On the mark pass, fish were given a caudal fin punch for identification on the recapture pass. Floy tag presence/absence and number, TL (mm), and weight (g) were recorded for all rainbow trout captured on both passes.

Floy-tagged fish captured in 2008 through 2011 were identified as GR×CRRs and were therefore included in the survival, growth, and abundance analyses. However, Floy tag loss occasionally prevented individual identification of GR×CRRs, precluding their inclusion in the survival analysis. Rainbow trout missing a Floy tag but retaining an adipose clip were identified as GR×CRRs for the purpose of abundance estimation, but were not included as part of the survival or growth analyses. In addition to the GR×CRRs, CRRs were present in the study section and were presumed to be remaining in the section from stocking events that occurred prior to the GR×CRR introduction in 2006. Rainbow trout from which a Floy tag and adipose clip were absent were identified as CRR, and CRR abundance was estimated separately from GR×CRR abundance for the years 2008, 2009, 2010, and 2011.

#### Survival estimation

Adult rainbow trout survival was estimated using data from recapture occasions occurring in the fall of 2006 and 2007, and the previously described abundance estimates of 2008, 2009, 2010, and 2011. Efforts in the fall of 2006 and 2007 consisted of two-pass removal estimates [21] conducted in a 305-m stretch of the upper Colorado River located at the upstream end of the study section (Figure 1). Estimates were completed using a four-electrode bank shocking unit and removal passes were conducted subsequently within the same day. Floy tag numbers, lengths, and weights were recorded for all GR×CRRs encountered during the sampling. Floy tag recaptures were used to estimate GR×CRR survival across all six years post-introduction, 2006 to 2011.

#### Statistical analyses

A Lincoln-Peterson estimator with a Bailey modification [22], which accounted for fish being returned to the population following examination of marks on the recapture pass [23], was used to obtain GR×CRR and CRR abundance estimates (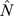) for each year of the study. Estimates were calculated for the entire study reach and divided by 4.2 (km sampled) to obtain an estimate of adult GR×CRR and CRR km^−1^ of river. Variance in abundance estimates was calculated using the equation presented in [23], and 95% confidence intervals (CIs) calculated from the variance estimates were used to compare differences in abundance between the GR×CRR and CRR within and across years.

Apparent survival probability (*φ*), the probability that fish survived and were retained within the study section, was estimated for the GR×CRR on a monthly basis, accounting for varying time intervals between primary sampling occasions, using the Cormack-Jolly-Seber (CJS) open capture-recapture estimator in Program MARK [24]. If tagged fish were encountered during either secondary sampling occasion (i.e., pass), the associated recapture data were used to create the encounter histories for the primary sampling occasions (fall 2006 and 2007, and spring 2008, 2009, 2010, and 2011). The model set included models in which detection probability (*p*) was constant, varied with discharge at time of sampling (cms), or varied by electrofishing method (to account for bank electrofishing occurring in the fall versus raft electrofishing occurring in the spring), or the additive combination of cms and electrofishing method. For survival estimation, the model set included models in which *φ* was constant, varied by length at release (length; included as an individual covariate), with minimum discharge between primary sampling occasions (min; Table 1), maximum discharge between primary sampling occasions (max; Table 1), or followed a trend with time (T). Although length was allowed to appear additively with min, max, or T, these three covariates never appeared in the same model. Models were ranked using Akaike’s Information Criterion corrected for small sample sizes (AICc) [25]. Model averaging was used to incorporate model selection uncertainty into the parameter estimates, and unconditional standard errors (SE) were reported for the model averaged parameter estimates [26].

**Table 1:** Minimum (min) and maximum (max) discharge values (cms) within primary study periods included as predictor variables affecting adult GR×CRR survival (*φ*).

Study Period	Min	Max
June 2006 - October 2006	1.06	14.52
October 2006 - October 2007	1.33	22.84
October 2007 - May 2008	2.22	21.23
May 2008 - April 2009	1.95	55.24
April 2009 - May 2010	1.96	39.22
May 2010 - May 2011	2.09	62.25

Absolute growth (TL) and absolute growth rate (TL year^−1^) of the GR×CRR were calculated using equations presented in [27]. Repeated measures of TL from individuals stocked in 2006 and recaptured between 2008 and 2011 were used to fit a von Bertalanffy growth curve by means of the Fabens method [28], where time at large (days), TL at release, and TL upon recapture were known. Time at large was converted from days to years prior to analysis, and parameters for the growth curve were estimated iteratively using a nonlinear regression approach [29] implemented in SAS (Proc NLIN) [30]. Age at recapture was calculated based on the knowledge that GR×CRRs were approximately 1.6 years of age at stocking. The von Bertalanffy model is a predictive model of growth, where growth rate declines with age, becoming zero as fish near a maximum possible size. The model is represented as 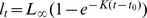, where 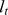 is length at time *t*, 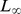 is the asymptotic length, *K* is a growth coefficient, and *t_0_* is a time coefficient at which length would theoretically be zero [31].

### Age-0 Trout Population

#### Population sampling

The age-0 (fry) population was sampled in September 2007 and October 2008 to determine the baseline genetic composition of the rainbow trout fry population produced in the upper Colorado River in these years. From 2009 to 2012, the salmonid fry population was sampled once a month, June through October, to determine fry abundance, as well as to determine if shifts in genetic composition of the rainbow trout fry population occurred over time. Three pass removal estimates were conducted using two LR-24 Smith-Root backpack electrofishing units run side-by-side to include all available fry habitat at four, 15.2 m-long sites, one located at the downstream end of the study section, two in the middle of the study section, and one at the upstream end of the study section (Figure 1).

All fry encountered during the sampling were identified to species, measured (TL; mm), and visually examined for signs of *M. cerebralis* infection. A fin clip was taken from all rainbow trout fry encountered during this sampling for genetic analysis. Additional electrofishing efforts outside of the population estimation sites were used to increase the number of the rainbow trout fry used in the genetic and disease (myxospore enumeration) analyses.

#### Genetic assignment of rainbow trout fry

The Genomic Variation Laboratory (GVL) at the University of California at Davis screened over 300 microsatellite markers and identified a suite of 18 markers capable of distinguishing pure GR and GR-cross fish, including GR×CRR (F1), second generation GR×CRR (F2), and backcross generations (B2C: F1 × CRR; B2G: F1 × GR), from pure CRR fish. This microsatellite panel included the following markers: BX310634, OMM5233, OMM1223, Omy1443, OMM1050, OMM5224, Omy1137INRA, OMM1008, OMM1238, OMM5262, Omy1090UW, OMM1118, Omy325UoG, OMM1076, OMM3072, OMM1082, OMM5149, and Omy1282INRA. PCR amplification and genotyping were conducted as previously described [32]. Known samples of pure GR, pure CRR, and their crosses, were used to identify microsatellites that were most effective for differentiation based on allele frequency differences between the pure strains; the ability of this microsatellite panel to differentiate simulated (hybridlab [33]) and blind samples was assessed prior to analysis of wild unknown samples.

The software programs NewHybrids [34] and Structure 2.3.3 [35] were used in tandem to differentiate pure strains and crosses. In NewHybrids, both uniform and Jeffreys-type priors were used along with specifying and not specifying pure individuals (z option) from known reference samples (i.e. four NewHybrids result files). Each Markov-Chain run had a burn-in period of 100,000 iterations followed by 1,000,000 iterations. An individual was positively identified as a specific strain or hybrid if the posterior probability for the given category was ≥80% for that individual. If none of the hybrid categories met this criterion, the individual was classified as unknown. In Structure, the number of genetic clusters (k) was set to two and the admixture model with correlated allele frequencies was used for two independent iterations with 100,000 burn-in and 500,000 Markov chain Monte Carlo (MCMC) repetitions. NewHybrid and Structure results were compared to ensure consistent individual assignment.

Rainbow trout fry collected from the upper Colorado River were genetically assigned to strain (pure GR, pure CRR) or cross (F1, F2, B2C, B2G). The proportion of the rainbow trout fry population assigned to the pure CRR or GR-cross hybrid categories, as well as classified as unknown, was ascertained on a per year basis, and trends across years were examined to determine if the GR×CRR had successfully reproduced in the upper Colorado River.

#### Quantification of M. cerebralis infection

Signs of infection as a result of exposure to *M. cerebralis*, including cranial, spinal, opercular, and lower jaw deformities, and blacktail, were recorded for each salmonid fry encountered between 2009 and 2012. In October of 2009 and 2011, brown trout fry (N = 60) and rainbow trout fry (N = 24) were collected from each of the four sites to quantify myxospores, a measure of the severity of infection following exposure to *M. cerebralis*. Myxospores were enumerated [36] using the pepsin-trypsin digest (PTD) method [37] by the Colorado Parks and Wildlife (CPW) Fish Health Laboratory (Brush, Colorado).

#### Statistical analyses

A three pass removal estimator [38] was used to obtain rainbow trout fry population abundance estimates (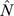) at each of the sampling sites. Estimates were converted to 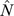 km^−1^ of river bank by multiplying the estimate by 65.8; estimates from the four sampling sites were averaged within a month, providing an estimate of fry km^−1^ of river bank for the entire study section. Confidence intervals [38] were used to compare differences in rainbow trout fry abundance both within and across years.

To evaluate the difference in myxospore counts of rainbow trout fry collected in 2009 and 2011, we used a general linear model (GLM) as implemented in SAS ProcGLM [30]; two models were included in the models set, an intercept-only model and a model including year as a categorical variable to capture inter-annual variation. The genetic assignment test was then used to associate myxospore count with rainbow trout fry determined to have CRR or GR-cross origins. A second GLM was run to examine if genotype conferred resistance to *M. cerebralis*, and if CRR and GR-cross fry differed from brown trout fry in average myxospore count. Two models were included in the model set, an intercept-only model, and a model including species as a categorical variable to capture inter-species variation. Logistic regression (SAS ProcLOGISTIC [30]) was used to assess the factors that influenced the probability that an individual fry would exhibit signs of *M. cerebralis* infection (cranial, spinal, opercular, and lower jaw deformities, and blacktail); disease sign was treated as a binary response variable (response was ‘yes’ or ‘no’). For the logistic regression analysis, we considered an intercept-only model, as well as models that included effects of species only, year only (2009, 2010, and 2011), and models with additive and interactive effects between species and year. Model weights and delta AICc ranking were used to determine support for each of the models included in the model sets, and parameter estimates were reported from the candidate model with the lowest AICc value [25].

## Results

### Adult Rainbow Trout Population

**Figure 2:**
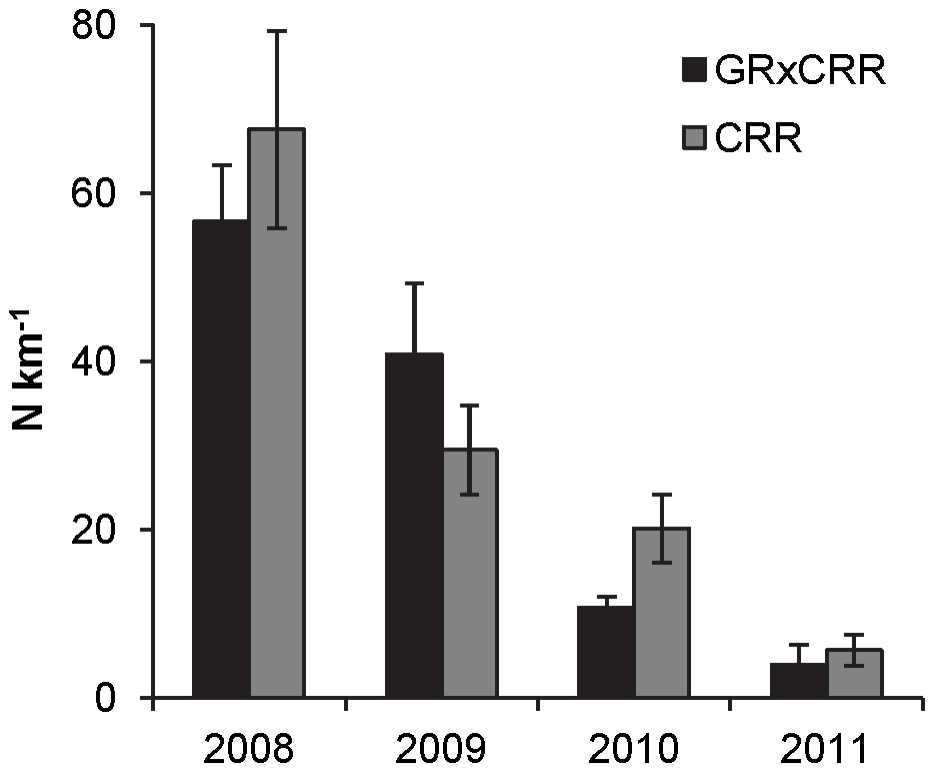
Adult GR×CRR and CRR abundance (N km^−1^; SE bars). Abundances were estimated within the upper Colorado River study section for the years 2008 to 2011.

Adult GR×CRR abundance (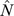 km^−1^; fish stocked in 2006 only) did not differ from adult CRR abundance in the upper Colorado River in any year. Both populations exhibited decreases in abundance between 2008 and 2011, declining from an estimated 57 (±8) GR×CRR and 68 (±15) CRR km^−1^ in 2008, to only 4 (±1) GR×CRR and 6 (±1) CRR km^−1^ in 2011 (Figure 2). Interestingly, the adult brown trout population also exhibited a decrease in abundance between 2009 and 2011, declining from an estimated 1,201 (±78) km^−1^ in 2009 to 525 (±47) km^−1^ in 2011.

**Figure 3:**
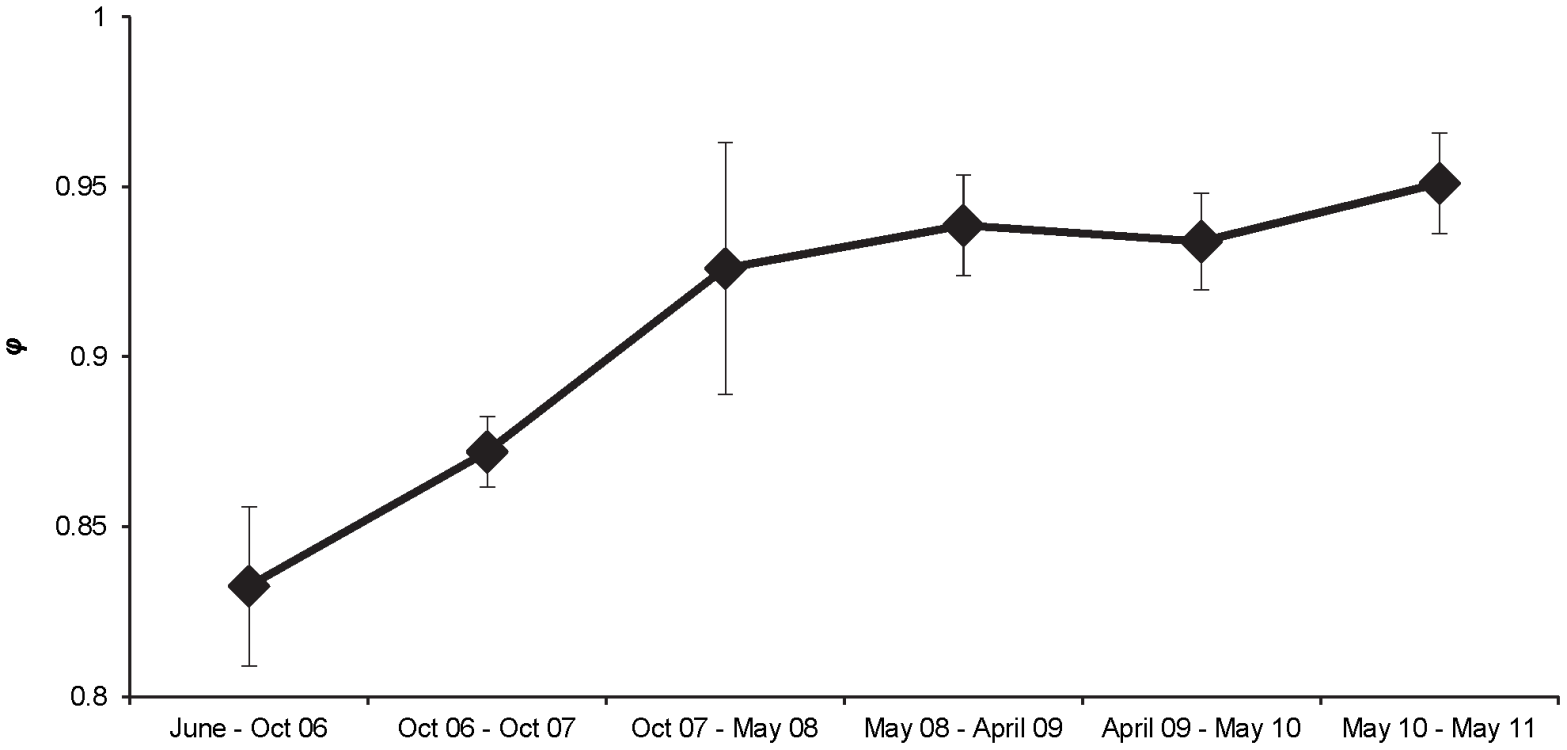
Model-averaged monthly apparent survival rate (φ; SE bars) for the GR×CRR. Survival rates apply only to the GR×CRR fish that were stocked in the upper Colorado River in June 2006. Date ranges (x-axis) represent the periods between primary sampling occasions for the adult rainbow trout population.

Apparent survival (*φ*) was more affected by discharge than a general trend with time. Models that allowed survival to vary as a function of minimum flow (top two models) between primary sampling occasions had twice as much support as those that modeled survival as a function of maximum flows (models ranked three and four; Table 2). Discharge had a positive effect on survival (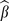 = 0.033±0.007), with survival increasing with an increase in minimum flow. Survival was also positively affected by length at release (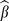 = 0.006±0.002), with length at release appearing in all six of the models with a ΔAICc value <4.0. In general, model-averaged monthly apparent survival was lower in 2006 and 2007 than it was in later years of the study (2008 through 2011; Figure 3), primarily due to minimum flows between primary sampling occasions that were nearly twice as low, on average, in 2006 and 2007 (1.21±0.13 cms) than in 2008 through 2011 (2.06±0.06 cms). Apparent survival for the entire study period (June 2006 to May 2011), the product of survival estimates within each study period, was estimated to be 0.007 (SE <0.001). Detection probability differed with electrofishing method (bank electrofishing *p = *0.05 [SE ±0.008]; raft electrofishing *p = *0.22 [SE ±0.06]), with electrofishing method appearing in all six models with a ΔAICc <4.0 (Table 2), and was likely due to the amount of stream length covered by the two sampling methods and the season in which sampling occurred. Discharge had a weak negative effect on *p* (associated 95% confidence intervals overlapped zero), and appeared in only three of the models with a ΔAICc value <4.0, and not in the top model.

**Table 2:** Model selection results for factors influencing apparent survival (*φ*) and detection probability (*p*) of the Floy tagged GR×CRR fish introduced to the upper Colorado River in June 2006.

Model^1^	log(*L*)	*K*	AICc	Δ*_i_*	*w_i_*
φ(L,MIN) *p*(E)	−873.51	5	1757.04	0.00	0.35
φ(L,MIN) *p*(E,CMS)	−872.82	6	1757.67	0.64	0.25
φ(L,MAX) *p*(E,CMS)	−873.27	6	1758.57	1.53	0.16
φ(L,MAX) *p*(E)	−874.63	5	1759.28	2.25	0.11
φ(L,T) *p*(E)	−875.14	5	1760.31	3.27	0.07
φ(L,T) *p*(E,CMS)	−874.27	6	1760.56	3.52	0.06
φ(MIN) *p*(E)	−881.02	4	1770.05	13.01	<0.01
φ(MIN) *p*(E,CMS)	−880.58	5	1771.18	14.14	<0.01
φ(L) *p*(E)	−881.72	4	1771.45	14.41	<0.01
φ(L) *p*(E,CMS)	−880.85	5	1771.73	14.69	<0.01
φ(MAX) *p*(E,CMS)	−881.00	5	1772.03	15.00	<0.01
φ(MAX) *p*(E)	−882.06	4	1772.13	15.09	<0.01
φ(T) *p*(E)	−882.51	4	1773.03	15.99	<0.01
φ(T) *p*(E,CMS)	−881.89	5	1773.81	16.77	<0.01
φ(L,MIN) *p*(CMS)	−882.29	5	1774.60	17.56	<0.01
φ(L) *p*(CMS)	−884.09	4	1776.20	19.16	<0.01
φ(L,T) *p*(CMS)	−883.40	5	1776.83	19.80	<0.01
φ(L,MAX) *p*(CMS)	−884.00	5	1778.03	20.99	<0.01

1 Models are ranked by their AICc differences (Δ*_i_*) relative to the best model in the set and Akaike weights (*w_i_*) quantify the probability that a particular model is the best model in the set given the data and the model set. Models for which there was weight are shown. Variables are: L = length, E = electrofishing method, CMS = discharge, MIN = minimum discharge between primary sampling occasions, MAX = maximum discharge between primary sampling occasions, and T = trend over time.

**Figure 4:**
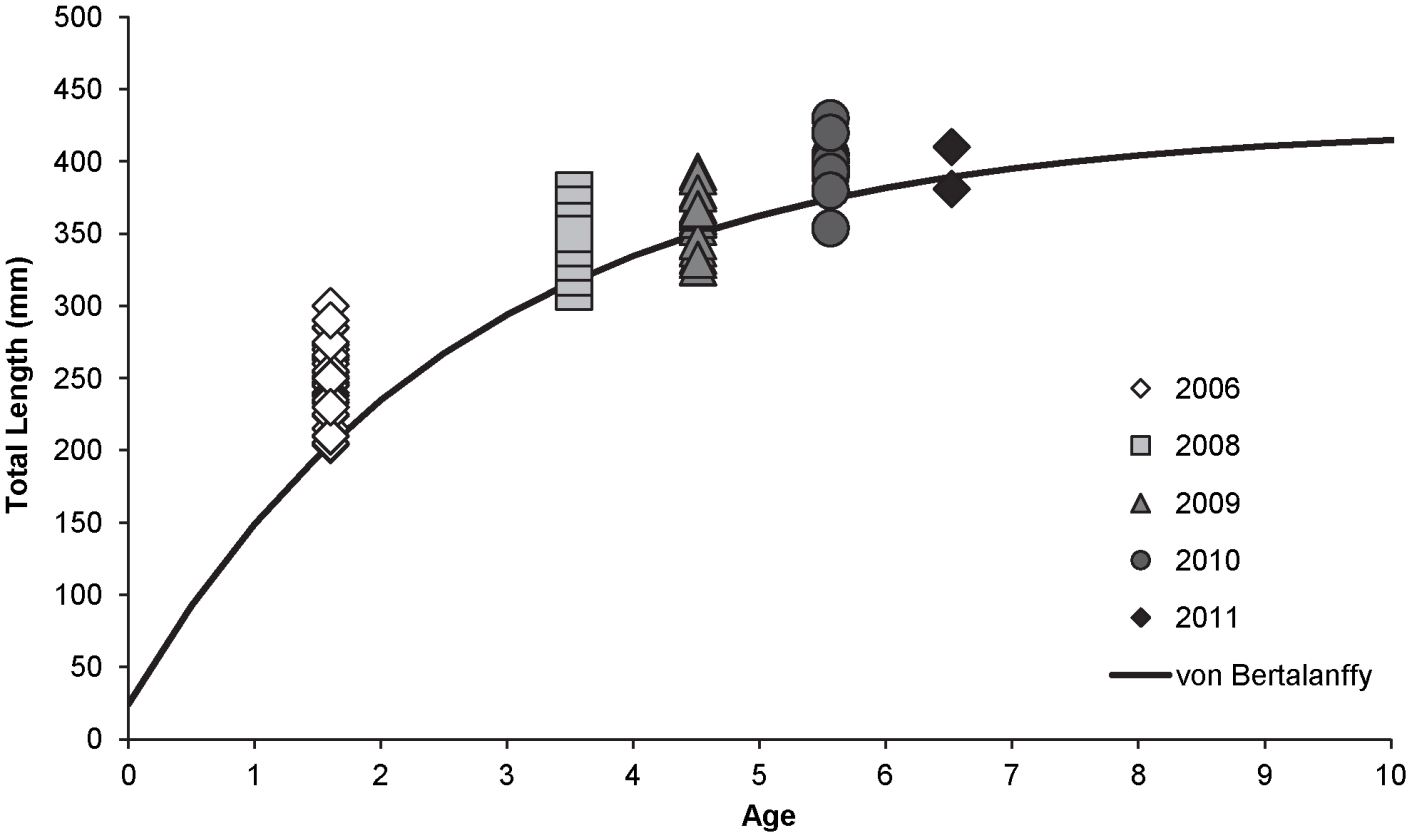
Predictive model of growth (TL; mm) trends of the GR×CRR stocked in the upper Colorado River in 2006. The von Bertalanffy growth function was determined using repeated measures of length from fish stocked in 2006 (1.6 years of age) and recaptured in 2008, 2009, 2010, or 2011.

Average absolute increase in TL (± SE) of the GR×CRR was 111 (±3.5) mm, with an average absolute annual rate of increase in TL of 45 (±1.3) mm. Parameter estimates for the von Bertalanffy equation were 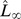 = 424.5, 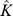 *= *0.37, and 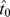 = −0.16 (Figure 4).

### Age-0 Trout Population

**Figure 5:**
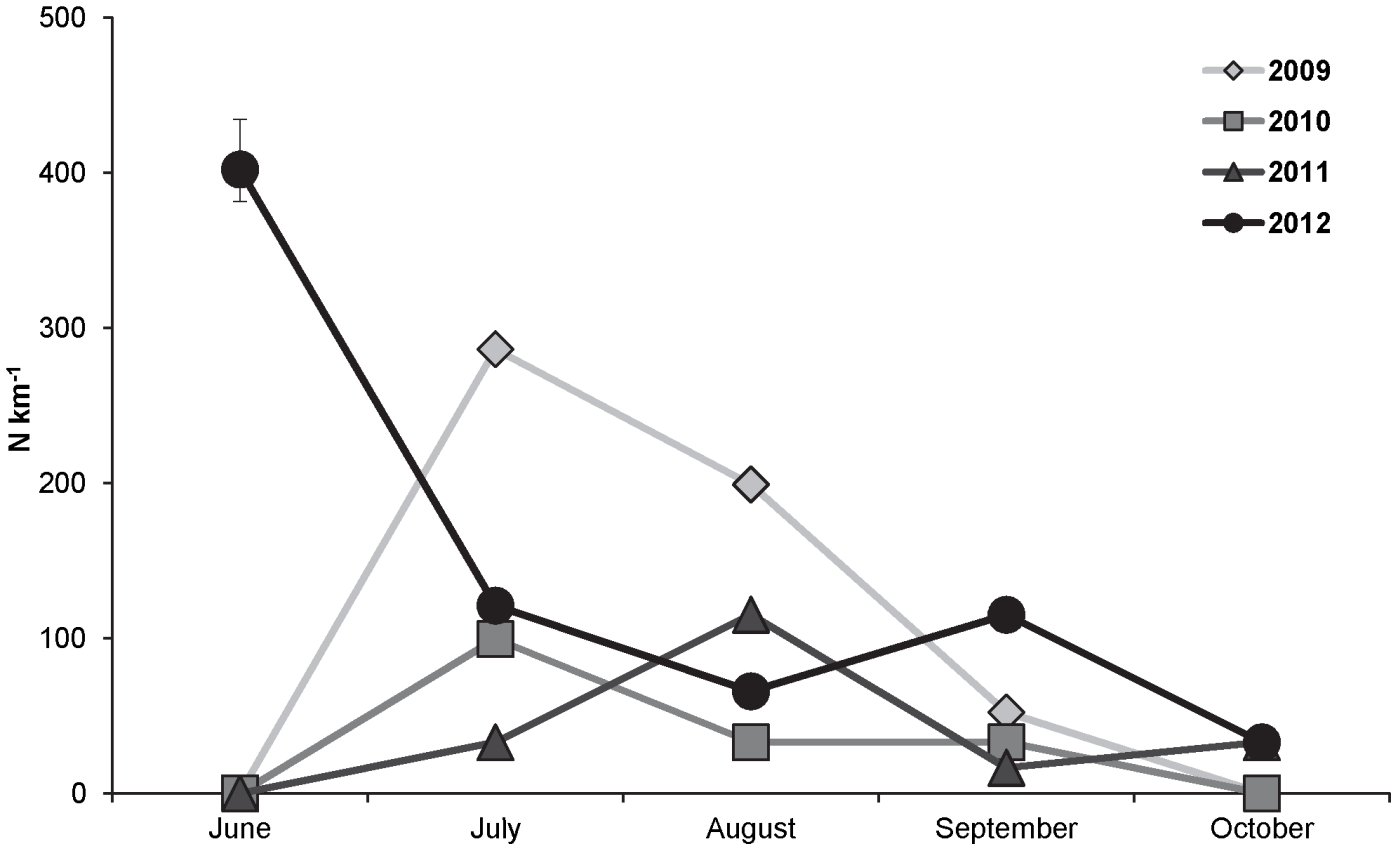
Rainbow trout fry abundance (N km^−1^; SE bars). Abundances were estimated within the upper Colorado River study section in June, July, August, September, and October of 2009, 2010, 2011, and 2012.

Wild rainbow trout fry abundance exhibited a declining trend between July and October in 2009 and 2010, and no rainbow trout fry were detected in any of the sampling sites in October of either year. A less exaggerated decreasing trend in rainbow trout fry abundance was observed in 2011 and 2012. Potentially indicative of an increase in resistance and survival, rainbow trout fry were still detected within the study sites in October of both 2011 and 2012 (Figure 5).

**Figure 6:**
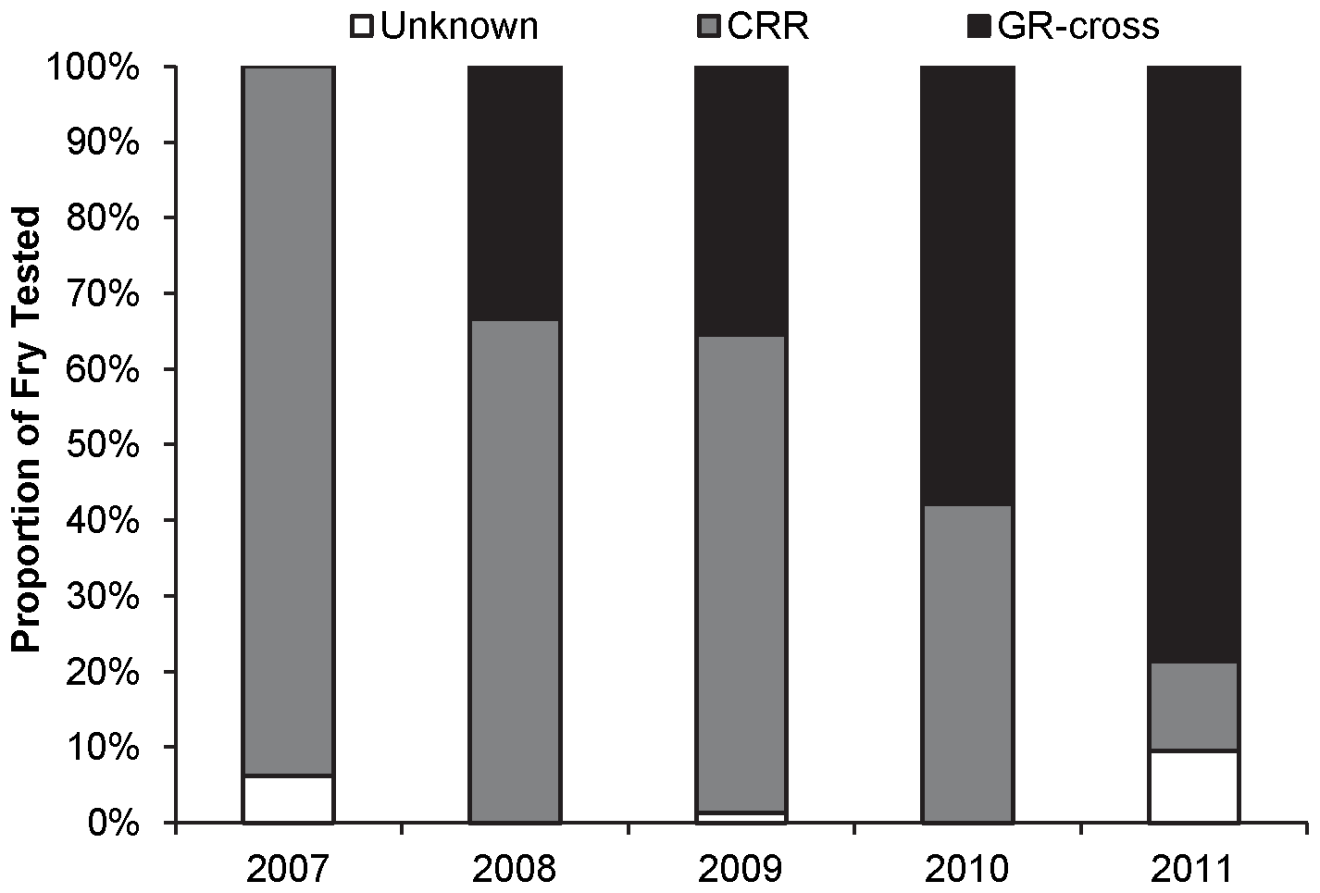
Proportion of the wild rainbow trout fry population assigned as CRR, GR-cross, or unknown. Fry were collected from the upper Colorado River in 2007 (N = 16), 2008 (N = 21), 2009 (N = 79), 2010 (N = 57), and 2011 (N = 42). Assignments were made when the posterior probability was ≥0.80 using the microsatellite marker genetic differentiation test.

Genetic assignments revealed a shift in the genetic composition of the rainbow trout fry population over time. In 2007, CRR and unknown fish comprised the entirety of the population (Figure 6). GR-cross fish first appeared in the fry population in 2008, comprising about 35% of the population. The proportion of GR-cross fish in the fry population increased over time, with GR-cross fish comprising nearly 80% of the fry population in 2011 (Figure 6). Of the GR-cross fish analyzed in 2011, 24% were identified as F1s, 36% as F2s, 24% as B2Cs, and 16% as B2Gs.

**Figure 7:**
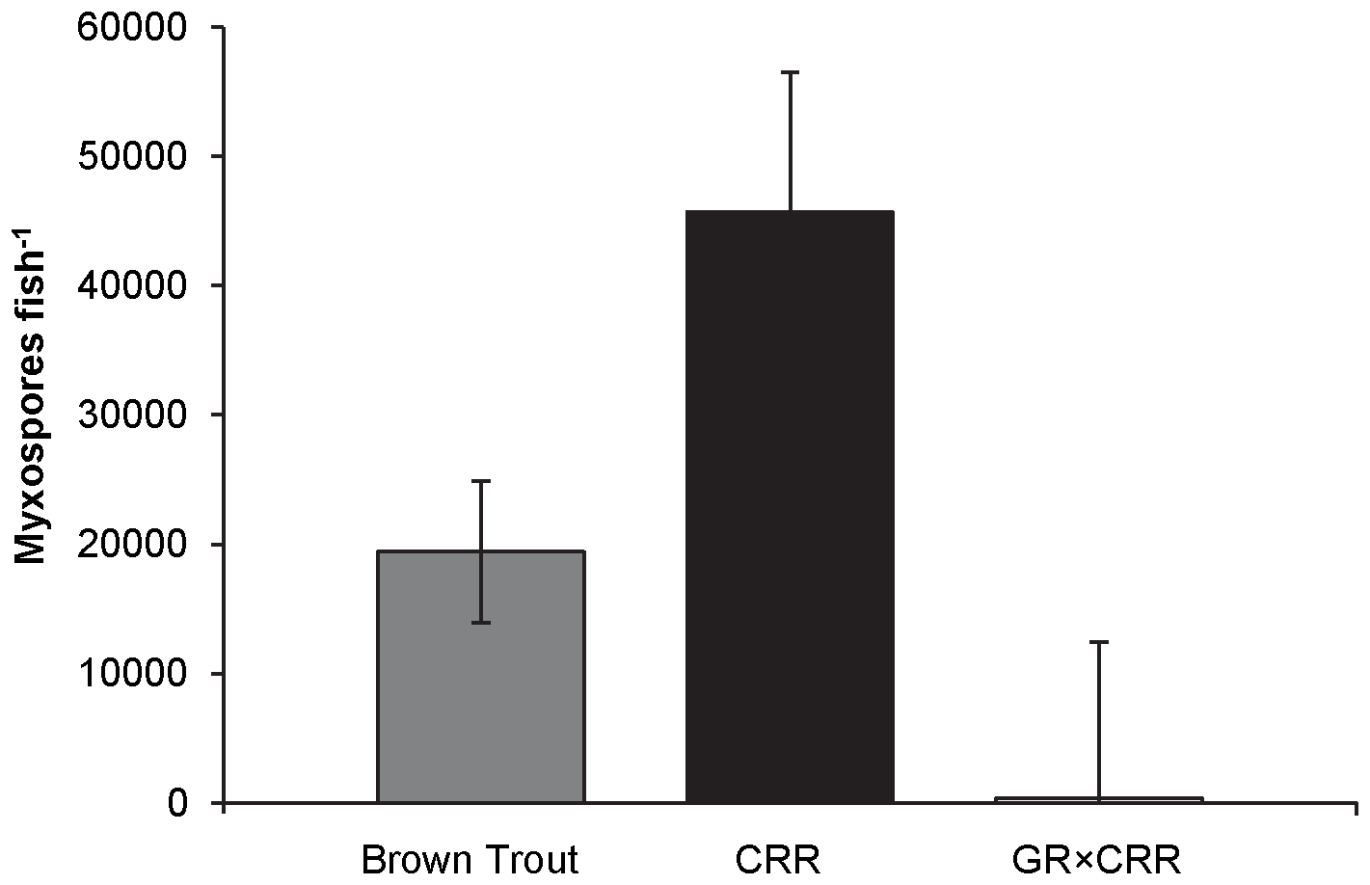
Average myxospore counts (myxospores fish^−1^; SE bars) of fry collected from the upper Colorado River. Brown trout (N = 60), CRR (N = 13), and GR×CRR (N = 11) fry were collected from the upper Colorado River in October of 2009 and 2011.

Model selection results for differences in average myxospore count in rainbow trout indicated that the model that included year was more supported by the data than the intercept model (AICc weight = 0.98). Fry collected in October of 2009 averaged 45,036 (±8,650) myxospores fish^−1^, whereas fry collected in October of 2011 (CRR and GR-cross) averaged 2,672 (±4,379) myxospores fish^−1^. When brown trout were included in the analysis and myxospore count was assigned to specific CRR or GR-cross rainbow trout individuals using the genetic assignment test, model selection results indicated that a model containing species/cross differences in myxospore count was most supported by the data (AICc weight = 0.93). CRR fry exhibited a higher myxospore count than either the GR-cross or brown trout fry (Figure 7).

**Figure 8:**
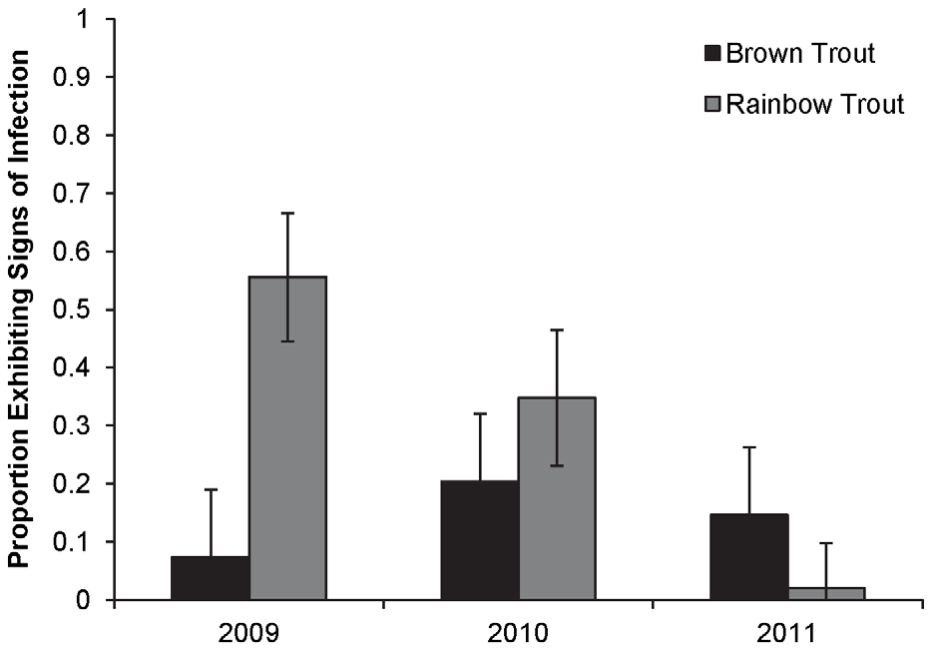
Proportion (SE bars) of the brown trout and rainbow trout fry populations exhibiting signs of *M. cerebralis* infection. Fry were collected from the upper Colorado River in 2009 (brown trout: N = 277; rainbow trout: N = 29), 2010 (brown trout: N = 64; rainbow trout: N = 41), and 2011 (brown trout: N = 138; rainbow trout: N = 19).

A species by year interaction had the largest influence on the probability that an individual fry would exhibit signs of *M. cerebralis* infection (AICc weight = 0.99; Table 3). A higher proportion of rainbow trout than brown trout fry exhibited signs of infection in 2009. No differences in the proportion of fish exhibiting signs of infection were observed between the two species in 2010 or 2011. The proportion of rainbow trout fry exhibiting signs of infection decreased between 2009 and 2011 (Figure 8), concurrent with the increase in the proportion of GR-cross fish in the fry population and decrease in infection severity (myxospores fish^−1^).

**Table 3:** Model selection results for factors influencing the probability that a fish exhibits signs of *M. cerebralis* infection in the upper Colorado River in the years 2009 through 2011.

Model^1^	*R* ^2^ ‡	log(*L*)	*K*	AICc	Δ*_i_*	*w_i_*
Species*Year	0.15	−214.06	6	445.06	0.00	0.99
Species+Year	0.10	−222.27	4	454.65	9.58	0.01
Species	0.08	−226.23	2	457.03	11.97	0.00
Year	0.06	−230.44	3	468.09	23.02	0.00
Intercept-only	0.00	−239.75	1	481.68	36.62	0.00

1 Models are ranked by their AICc differences (Δ*_i_*) relative to the best model in the set and Akaike weights (*w_i_*) quantify the probability that a particular model is the best model in the set given the data and the model set.

‡ *R* ^2^ values are maximum rescaled *R* ^2^ values.

## Discussion

Stocked adult rainbow trout exhibited low survival following stocking; however, they did reproduce. GR×CRR rainbow trout were stocked into our study section in 2006, and they began to reproduce in 2008. Before GR×CRR reproduction occurred, CRR individuals comprised the entire fry population due to stocking of this strain in the upper Colorado River prior to the 2006 introduction of the GR×CRR fish. However, subsequent age-0 sampling indicated that GR-cross genotypes were increasing in prevalence relative to the CRR strain. Interestingly, we observed the first age-0 recruitment into October in 2011 and 2012. Age-0 rainbow trout exhibited an increase in resistance characteristics over time as a result.

As resistant genotypes increased, average infection severity (myxospores fish^−1^) and percentage of age-0 exhibiting signs of exposure to *M. cerebralis* decreased. The myxospore counts of age-0 fish collected in 2009 were similar to those obtained from age-0 rainbow trout collected in the upper Colorado River from about 1990 to 2000 and were indicative of infection levels that caused the original decline [7], [16]. Myxospore counts of fish collected in 2011 were significantly lower than most myxospore counts observed in earlier studies [39], [40] and as low as those observed for brown trout. *M. cerebralis* is endemic in brown trout from central Europe to southeastern Asia and does not cause disease in these populations [41]. Similarly, GR strain fish developed resistance to *M. cerebralis* in a German fish hatchery [11]. In the upper Colorado River, age-0 GR-cross fish did not differ in infection severity from the age-0 brown trout, suggesting that they were just as resistant to infection and the on-set of clinical signs as the brown trout.

Age-0 CRR had a two-fold and 127-fold higher myxospore count, on average, than either the brown trout or GR-cross fry, respectively, and this is consistent with other studies showing that CRR are highly susceptible to *M. cerebralis* infection [9], [12], [40]. Myxospore levels in CRR individuals indicate that the parasite is still prevalent in the upper Colorado River and that the low myxospore levels in the GR-cross are not a result of reduced parasite numbers. Although differences in myxospore count were previously observed during laboratory experiments [9], [12], our field observations are the first to document such differences in wild populations. Reduced myxospore burdens in age-0 GR-cross trout indicate that stocking this cross may ultimately lead to an overall reduction in infection prevalence and severity in the salmonid populations of the upper Colorado River.

Recruitment of age-0 fish into October, observed in 2011 and 2012, was associated with the shift in genetic composition and decrease in infection severity. Prior to 2011, age-0 rainbow trout quickly developed clinical signs and were not observed in the river by October [7], [16]. We attribute the lack of recruitment to low survival in the younger age classes following exposure to *M. cerebralis* and this is supported by *in situ* studies conducted in the same area [7]. Survival of rainbow trout fry into October of 2011 and 2012 suggests that GR-cross rainbow trout fry produced in the river may be better able to survive exposure to *M. cerebralis* than their wild CRR counterparts, and that natural recruitment may soon start to aid in the recovery of the wild rainbow trout population in the upper Colorado River.

Fetherman et al. [12] suggest that resistance to *M. cerebralis* is a heritable trait that should respond to natural selection in the wild. Therefore, continued exposure to *M. cerebralis* in the wild should favor retention of resistance traits, increasing the probability of their persistence. Resistance to *M. cerebralis* in a similar rainbow trout population from Harrison Lake, Montana has increased with continued exposure to the parasite [42]. Miller and Vincent [42] suggest that as more resistant young from the population mature and reproduce, it may be possible for the population to return to abundance levels observed prior to parasite establishment. Although recovery of wild rainbow trout populations in Colorado was expected to be relatively slow given the low survival of *M. cerebralis* infected fish in wild CRR populations [43], the introduction of resistant GR-crosses may facilitate quicker recovery of these populations [12].

Apparent survival was low in stocked GR×CRR rainbow trout. The hatchery derived origin and history of domestication selection for growth and resistance in the GR strain may have contributed to the low survival rates observed in the reintroduced GR×CRR population; the GR strain is also known to exhibit low heterozygosity [44] which may be an issue with stocked GR×CRR populations. In addition, research has shown that the GR strain and high proportion GR-crosses (≥0.75; e.g., B2G) exhibit lower survival and increased predation susceptibility compared to CRR when introduced to natural systems with many terrestrial predators and piscivorous fish species [45]. Despite potential drawbacks associated with the resistant, domestic GR strain, laboratory experiments confirmed that GR×CRR exhibited a higher resistance to *M. cerebralis* relative to the susceptible, wild CRR strain, and that critical swimming velocities did not differ from that of the CRR strain [13]. Therefore, the GR×CRR was expected to be better suited for survival in the upper Colorado River than either parental strain.

Survival was also influenced by environmental factors, particularly flow. Both GR×CRR and wild brown trout populations exhibited similar population declines over the study period suggesting that environmental conditions may have influenced GR×CRR survival, and results suggest that minimum discharge had a large negative effect on GR×CRR survival. Lower flows result in higher summer water temperatures and lower dissolved oxygen levels [46], both of which can directly affect salmonid survival [47]. Increased stress due to low flow may have also intensified the effects of *M. cerebralis* infection. Ectoparasite infestation peaks during periods of low flow and high mean water temperatures in the upper Colorado River and could significantly increase mortality in these populations [18]. Low flows also reduce suitable habitat and can lead to high densities and overcrowding, increased predation, and increased competition [48]. Brown trout competition with rainbow trout results in exclusion of rainbow trout from preferred feeding and resting habitats, possibly resulting in population level effects with respect to abundance and survival [49].

Availability of food resources may also influence reintroduction efforts. The upper Colorado River below Windy Gap Reservoir has undergone significant changes to aquatic invertebrate diversity and abundance; in particular the abundance of the giant stonefly (*Pteronarcys californica)* has significantly decreased in recent years [50]. We believe that differences in prey diversity, abundance and size may explain current adult rainbow trout size and differences with historic rainbow trout size. Our von Bertalanffy modeling and parameter estimates provide the first description of growth for *M. cerebralis*-resistant rainbow trout in a natural system. Maximum asymptotic length (424.5 mm) is similar to maximum lengths observed in brown trout during the study (CPW, unpublished data). However, prior to the introduction of *M. cerebralis*, rainbow trout (CRR) and brown trout greater than 425 mm were consistently observed during annual population estimates [7]. Laboratory experiments indicate that GR×CRR fish grew faster and were significantly larger than CRR fish of the same age [13] and we predicted that GR×CRR fish would attain larger sizes than those observed in the pre-*M. cerebralis* CRR population. We believe that differences in fish length pre- and post-*M. cerebralis* introduction are, at least in part, due to changes in food resources rather than *M. cerebralis* infection or strain performance differences.

## Conclusions

Fraser [5] suggested that a successful reintroduction of salmonids may take 15 to 20 years or longer. Reintroduction of a self-sustaining population of rainbow trout in the upper Colorado River has been influenced by environmental conditions as well as disease presence, and success will likely depend on both favorable environmental conditions and increased resistance to *M. cerebralis*. Although the rainbow trout population in the upper Colorado River is showing signs of recovery, it has not yet become a self-sustaining population [5]. Our results suggest that supplemental stocking will be needed for continued persistence in the upper Colorado River; however, age-0 results clearly show that resistant fish reproduced, and that their offspring survived at least until the fall in the upper Colorado River. The survival of age-0 fish to the fall suggests that recruitment may be forthcoming. However, lack of recruitment continues to contribute to the decline in the adult rainbow trout population in the upper Colorado River. Recruitment may have occurred in 2012 as age-0 rainbow trout were still present in October 2011; low water prevented population evaluation in the spring of 2012.

We suggest that artificial supplementation and annual monitoring of the rainbow trout population should continue to evaluate whether our observed survival of age-0 fish is followed by subsequent recruitment to the adult reproductive population. Future management should focus on increasing adult rainbow trout survival and retention in locations where GR×CRR are reintroduced. Such management strategies may include brown trout removal or habitat modifications. Additional introduction strategies should be evaluated, such as introducing large numbers of smaller GR×CRR. We believe that the introduction of *M. cerebralis*-resistant rainbow trout remains a promising management strategy for the reintroduction of rainbow trout fisheries in Colorado and elsewhere.

## References

[1] Hesthagen T, Larsen BM (2003) Recovery and re-establishment of Atlantic salmon, *Salmo salar*, in limed Norwegian rivers. Fisheries Manag Ecol 10: 87–95 10.1046/j.13652400.2003.00326.x

[2] Flagg TA, McAuley MC, Kline PA, Powell MS, Taki D, et al (2004) Application of captive broodstocks to preservation of ESA-listed stocks of Pacific salmon: Redfish Lake Sockeye Salmon case example. Am Fish Soc Symp 44: 387–400.

[3] Bosch WJ, Newsome TH, Dunnigan JL, Hubble JD, Neeley D (2007) Evaluating the feasibility of reestablishing a coho salmon population in the Yakima River, Washington. N Am J Fish Manage 27: 198–214 10.1577/M05044.1

[4] Carmona-Catot G, Moyle PB, Simmons RE (2012) Long-term captive breeding does not necessarily prevent reestablishment: lessons learned from Eagle Lake rainbow trout. Rev Fish Biol Fisher 22: 325–342 10.1007/s111600119230-x

[5] Fraser DJ (2008) How well can captive breeding programs conserve biodiversity? A review of salmonids. Evol Appl 1: 535–586 10.1111/j.17524571.2008.00036.x 25567798 PMC3352391

[6] Harig AL, Fausch KD (2002) Minimum habitat requirements for establishing translocated cutthroat trout populations. Ecol Appl 12: 535–551 10.2307/3060961

[7] Nehring RB, Thompson KG (2001) Impact assessment of some physical and biological factors in the whirling disease epizootic among wild trout in Colorado. Denver: Colorado Division of Wildlife Aquatic Research Special Report Number 76. 78 p.

[8] Thompson KG (2011) Evaluation of small-scale habitat manipulation to reduce the impact of the whirling disease parasite in streams. Aquat Ecosyst Health Manag 14: 305–317 10.1080/14634988.2011.602276

[9] Schisler GJ, Myklebust KA, Hedrick RP (2006) Inheritance of *Myxobolus cerebralis* resistance among F1-generation crosses of whirling disease resistant and susceptible rainbow trout strains. J Aquat Anim Health 18: 109–115 10.1577/H05047.1

[10] Halverson A (2010) An entirely synthetic fish: how rainbow trout beguiled America and overran the world. New Haven: Yale University Press. 257 p.

[11] Hedrick RP, McDowell TS, Marty GD, Fosgate GT, Mukkatira K, et al (2003) Susceptibility of two strains of rainbow trout (one with suspected resistance to whirling disease) to *Myxobolus cerebralis* infection. Dis Aquat Org 55: 37–44 10.3354/dao055037 12887253

[12] Fetherman ER, Winkelman DL, Schisler GJ, Antolin MF (2012) Genetic basis of differences in myxospore count between whirling disease-resistant and -susceptible strains of rainbow trout. Dis Aquat Org 102: 97–106 10.3354/dao02543 23269384

[13] Fetherman ER, Winkelman DL, Schisler GJ, Myrick CA (2011) The effects of *Myxobolus cerebralis* on the physiological performance of whirling disease resistant and susceptible strains of rainbow trout. J Aquat Anim Health 23: 169–177 10.1080/08997659.2011.630273 22372244

[14] Walker PG, Nehring RB (1995) An investigation to determine the cause(s) of the disappearance of young wild rainbow trout in the Upper Colorado River, in Middle Park, Colorado. Denver: Colorado Division of Wildlife Report. 134 p.

[15] USGS (2009) USGS real time water data for the nation. Available: <http://nwis.waterdata.usgs.gov/co/nwis/uv/?>. Accessed 2009 July 17.

[16] Nehring RB (2006) Colorado’s cold water fisheries: whirling disease case histories and insights for risk management. Denver: Colorado Division of Wildlife Aquatic Wildlife Research Special Report Number 79. 46 p.

[17] Schisler GJ, Bergersen EP, Walker PG (1999a) Evaluation of chronic gas supersaturation on growth, morbidity, and mortality of fingerling rainbow trout infected with *Myxobolus cerebralis* . N Am J Aquacult 61: 175–183 doi: –;10.1577/1548–8454(1999)061<0175:EOCGSO>2.0.CO;2

[18] Schisler GJ, Walker PG, Chittum LA, Bergersen EP (1999b) Gill ectoparasites of juvenile rainbow trout and brown trout in the upper Colorado River. J Aquat Anim Health 11: 170–174 doi: –;10.1577/1548–8667(1999)011<0170:GEOJRT>2.0.CO;2

[19] Schisler GJ, Bergersen EP, Walker PG (2000) Effects of multiple stressors on morbidity and mortality of fingerling rainbow trout infected with *Myxobolus cerebralis* . T Am Fish Soc 129: 859–865 doi: –;10.1577/1548–8659(2000)129<0859:EOMSOM>2.3.CO;2

[20] Ryce EKN, Zale AV, MacConnell E, Nelson M (2005) Effects of fish age versus size on the development of whirling disease in rainbow trout. Dis Aquat Org 63: 69–76 10.3354/dao059225 15759802

[21] Temple GM, Pearsons TN (2007) Electrofishing: backpack and drift boat. In: Johnson DH, Schrier BM, O’Neal JS, Knutzen JA, Augerot X, O’Neal TA, Pearsons TN, editors. Salmonid field protocols handbook: techniques for assessing status and trends in salmon and trout populations. Bethesda: American Fisheries Society. pp. 95–132.

[22] Bailey NTJ (1951) On estimating the size of mobile populations from recapture data. Biometrika 38: 293–306 10.1093/biomet/38.34.293

[23] Van Den Avyle MJ, Hayward RS (1999) Dynamics of exploited fish populations. In: Kohler CG, Hubert WA, editors. Inland fisheries management in North America. Bethesda: American Fisheries Society. pp. 127–166.

[24] White GC, Burnham KP (1999) Program MARK: Survival estimation from populations of marked animals. Bird Study 46 Supplement: 120–138 10.1080/00063659909477239

[25] Burnham KP, Anderson DR (2002) Model selection and multimodel inference: a practical information-theoretic approach. New York: Springer-Verlag. 488 p.

[26] Anderson DR (2008) Model based inference in the life sciences: a primer on evidence. New York: Springer, LLC. 184 p.

[27] Busacker GP, Adelman IR, Goolish EM (1990) Growth. In: Schreck CB, Moyle PB, editors. Methods for fish biology. Bethesda: American Fisheries Society. pp. 363–387.

[28] Fabens AJ (1965) Properties and fitting of the von Bertalanffy growth curve. Growth 29: 265–289. PubMed: 5865688. 5865688

[29] Isely JJ, Grabowski TB (2007) Age and growth. In: Guy CS, Brown ML, editors. Analysis and interpretation of freshwater fisheries data. Bethesda: American Fisheries Society. pp. 187–228.

[30] SAS Institute, Inc (2010) SAS system software, release 9.2. Cary: SAS Institute, Inc.

[31] Bertalanffy L (1938) A quantitative theory of organic growth (inquiries on growth laws II). Hum Bio 10: 181–213.

[32] Baerwald MR, Petersen JL, Hedrick RP, Schisler GJ, May B (2011) A major effect quantitative trait locus for whirling disease resistance identified in rainbow trout (*Oncorhynchus mykiss*). Heredity 106: 920–926 10.1038/hdy.2010.137 21048672 PMC3186244

[33] Nielsen EE, Hansen MM, Bach LA (2001) Looking for a needle in a haystack: discovery of indigenous Atlantic salmon (*Salmo salar* L.) in stocked populations. Conserv Genet 2: 219–232 10.1023/A:1012239029574

[34] Anderson EC, Thompson EA (2002) A model-based method for identifying species hybrids using multilocus genetic data. Genetics 160: 1217–1229. PubMed: 11901135. 10.1093/genetics/160.3.1217 PMC1462008 11901135

[35] Pritchard JK, Stephens M, Donnelly P (2000) Inference of population structure using multilocus genotype data. Genetics 155: 945–959. PubMed: 10835412. 10.1093/genetics/155.2.945 PMC1461096 10835412

[36] O’Grodnick JJ (1975) Whirling disease *Myxosoma cerebralis* spore concentration using the continuous plankton centrifuge. J Wildl Dis 11: 54–57. PubMed: 803578. 10.7589/0090-3558-11.1.54 803578

[37] Markiw ME, Wolf K (1974) *Myxosoma cerebralis*: isolation and concentration from fish skeletal elements – sequential enzymatic digestions and purification by differential centrifugation. J Fish Res Board Can 31: 15–20.

[38] Seber GAF, Whale JF (1970) The removal method for two and three samples. Biometrics 26: 393–400. PubMed: 5480657. 5480657

[39] Thompson KG, Nehring RB, Bowden DC, Wygant T (1999) Field exposure of seven species of subspecies of salmonids to *Myxobolus cerebralis* in the Colorado River, Middle Park, Colorado. J Aquat Anim Health 11: 312–329 doi: –;10.1577/1548–8667(1999)011<0312:FEOSSO>2.0.CO;2

[40] Ryce EKN, Zale AV, Nehring RB (2001) Lack of selection for resistance to whirling disease among progeny of Colorado River rainbow trout. J Aquat Anim Health 12: 63–68 doi: –;10.1577/1548–8667(2001)013<0063:LOSFRT>2.0.CO;2

[41] Granath WO, Gilbert MA, Wyatt-Pescador EJ, Vincent ER (2007) Epizootiology of *Myxobolus cerebralis*, the causative agent of salmonid whirling disease in the Rock Creek drainage of west-central Montana. J Parasitol 93: 104–119. PubMed: 17436949. 10.1645/GE-948R.1 17436949

[42] Miller MP, Vincent ER (2008) Rapid natural selection for resistance to an introduced parasite of rainbow trout. Evol Appl 1: 336–341 10.1111/j.17524571.2008.00018.x 25567635 PMC3352428

[43] Nehring RB, Thompson KG (2003) Whirling disease risk assessment: the Colorado perspective. In: Whirling Disease Initiative, editor. Managing the risk: proceedings of the 9^th^ annual whirling disease symposium. Bozeman: Whirling Disease Initiative. pp. 31–32.

[44] El-Matbouli M, Oucible A, Severin V, Meyer U, Grabner D, et al. (2006) Data on the mechanisms associated with the resistance of Hofer- and wild rainbow trout strains to whirling disease. In: Whirling Disease Initiative, editor. War of the whirlds: proceedings of the 12^th^ annual whirling disease symposium. Bozeman: Whirling Disease Initiative. pp. 22–23.

[45] Fetherman ER, Schisler GJ (2012) Sport fish research studies. In: Anonymous, editor. Federal Aid in Fish and Wildlife Restoration, job progress report. Fort Collins: Colorado Parks and Wildlife, Aquatic Wildlife Research Section. 68 p.

[46] Williams JE, Haak AL, Neville HM, Colyer WT (2009) Potential consequences of climate change to persistence of cutthroat trout populations. N Am J Fish Manage 29: 533–548 10.1577/M08072.1

[47] Hicks BJ, Beschta RL, Harr RD (1991) Long-term changes in streamflow following logging in western Oregon and associated fisheries implications. Water Resour Bull 27: 217–226 10.1111/j.17521688.1991.tb03126.x

[48] Arismendi I, Safeeq M, Johnson SL, Dunham JB, Haggerty R (2012) Increasing synchrony of high temperature and low flow in western North America streams: double trouble for coldwater biota? Hydrobiologia (2012): 1–10 10.1007/s1075001213272

[49] Gatz AJ, Sale MJ, Loar JM (1987) Habitat shifts in rainbow trout: competitive influences of brown trout. Oceologia 74: 7–19 10.1007/BF00377339 28310408

[50] Nehring RB, Heinold B, Pomeranz J (2011) Colorado River aquatic resources investigations. In: Anonymous, editor. Federal Aid in Fish and Wildlife Restoration, job progress report. Fort Collins: Colorado Division of Wildlife, Aquatic Wildlife Research Section.

